# Staphylococcus epidermidis Bacteremia in an Older Patient With Guillain-Barré Syndrome With Fever of Unknown Origin: A Case Report

**DOI:** 10.7759/cureus.45940

**Published:** 2023-09-25

**Authors:** Tomoe Nagayama, Junya Ohara, Chiaki Sano, Ryuichi Ohta

**Affiliations:** 1 Family Medicine, International University of Health and Welfare Graduate School of Health Sciences, Tokyo, JPN; 2 Family Medicine, Unnan City Hospital, Unnan, JPN; 3 Community Medicine Management, Shimane University Faculty of Medicine, Izumo, JPN; 4 Communiy Care, Unnan City Hospital, Unnan, Shimane, JPN

**Keywords:** general medicine, respiratory muscle paralysis, staphylococcus epidermidis, older, family medicine, rural, japan, immunosuppression, guillain–barré syndrome

## Abstract

Guillain-Barré syndrome (GBS) is an immune-mediated disorder that affects the peripheral nerves, often leading to weakness, numbness, and paralysis. Although GBS does not induce immunosuppression, severe cases can render patients vulnerable to infection due to various complications. We present the case of a 70-year-old woman who developed GBS following a *Mycoplasma *infection. The patient’s prolonged GBS symptoms led to an immunocompromised state, resulting in sepsis due to bacteremia caused by methicillin-resistant *Staphylococcus epidermidis*. Respiratory muscle paralysis necessitated intubation and mechanical ventilation, predisposing the patient to aspiration pneumonia. Prolonged hospitalization increases the risk of infection, as exemplified by catheter-related bloodstream infections and respiratory bacterial colonization. Although GBS does not inherently suppress immunity, its complications, such as musculoskeletal and respiratory failure, can mimic immunodeficiency, necessitating comprehensive management. A system-based approach should address neurological deficits and potential complications, emphasizing collaboration among medical specialties. This case highlights the importance of recognizing GBS-related challenges and adopting a holistic strategy for effective patient care.

## Introduction

Guillain-Barré syndrome (GBS) is a condition in which the immune system attacks peripheral nerves, leading to symptoms ranging from weakness and numbness to paralysis in severe cases [1]. The direct effects of GBS are related to nerve damage rather than immunosuppression. However, indirect ways GBS can render a person more susceptible to infection exist and be caused by systemic failures such as respiratory and musculoskeletal systems [2]. Severe GBS can cause respiratory muscle weakness, leading to respiratory failure [2]. Therefore, these patients may require mechanical ventilation [2]. Being on a ventilator, especially for an extended period, can increase the risk of respiratory infections, including pneumonia [3]. Prolonged hospital stays, which can occur in severe GBS, are associated with an increased risk of nosocomial infections, including urinary tract infections from catheters and bloodstream infections [4]. Patients with GBS may be bedridden or have limited mobility for extended periods, which can lead to complications such as bedsores or deep vein thrombosis [4]. Distinguishing between increased susceptibility to complications and true immunosuppression, wherein the ability of the immune system to respond to pathogens is compromised, is crucial.

Herein, we report a case where prolonged GBS symptoms led to an immunocompromised state following a Mycoplasma infection. This subsequently resulted in sepsis due to bacteremia caused by methicillin-resistant coagulase-negative *Staphylococcus epidermidis*. Moreover, during the course of the respiratory muscle and vocal cord paralysis, the patient vomited repeatedly, indicating that she had aspirated Citrobacter, an intestinal bacterium. While many GBS cases are mild and resolve naturally, progression to a severe state can lead to prolonged paralytic symptoms that cause immunosuppression, potentially leading to fever of unknown origin and bacteremia from unspecified sources [5]. This case report discusses the need to consider and manage the potential for immunodeficiency and associated infections due to severe GBS progression.

## Case presentation

A 70-year-old woman, previously performing her activities of daily living independently, presented to our facility with an acute onset of lower limb muscle weakness that had started three days before the presentation. Ten days before her visit, she developed a dry cough that progressively worsened. Three days before her appointment, she began experiencing difficulty exerting strength throughout her body. On the day of her presentation, she could not stand or perform daily activities, leading to an emergency transfer to our hospital. She had no fever, chills, nausea, vomiting, or diarrhea. She had no significant medical history and was not on any medications.

Upon examination, her vital signs were as follows: temperature of 35.1 ℃, pulse of 66 bpm, respiration rate of 13/min, blood pressure of 144/90 mmHg, and peripheral oxygen saturation of 95% on room air. A neurological examination revealed no abnormalities in the cranial nerves. Manual muscle testing revealed scores of 5/5 for the right and left trapezius, biceps, triceps, wrist extensors, and wrist flexors; 4/4 for the iliopsoas, quadriceps, and hamstring muscles; and 3/3 for the anterior tibialis and gastrocnemius muscles. Tendon reflexes were diminished in the lower extremities, and glove-and-stocking-type sensory deficits were noted. Tendon reflexes were reduced in all four limbs, and no contraction of the anal sphincter was noted. Initial laboratory data revealed mild inflammation (Table 1).

**Table 1:** Initial laboratory data of the patient. eGFR: estimated glomerular filtration rate; CRP: C-reactive protein; SARS-CoV-2: severe acute respiratory syndrome coronavirus 2.

Parameters	Level	Reference
White blood cells	9.8	3.5–9.1 × 10^3^/μL
Neutrophils	90.2	44.0–72.0%
Lymphocytes	7.4	18.0–59.0%
Monocytes	1.9	0.0–12.0%
Eosinophils	0.1	0.0–10.0%
Basophils	0.4	0.0–3.0%
Red blood cells	5.11	3.76–5.50 × 10^6^/μL
Hemoglobin	16.6	11.3–15.2 g/dL
Hematocrit	49.8	33.4–44.9%
Mean corpuscular volume	97.3	79.0–100.0 fL
Platelets	22.2	13.0–36.9 × 10^4^/μL
Total protein	7.7	6.5–8.3 g/dL
Albumin	4.5	3.8–5.3 g/dL
Total bilirubin	0.9	0.2–1.2 mg/dL
Aspartate aminotransferase	21	8–38 IU/L
Alanine aminotransferase	17	4–43 IU/L
Alkaline phosphatase	147	106–322 U/L
γ-Glutamyl transpeptidase	25	<48 IU/L
Lactate dehydrogenase	227	121–245 U/L
Blood urea nitrogen	12.3	8–20 mg/dL
Creatinine	0.54	0.40–1.10 mg/dL
eGFR	83.1	>60.0 mL/min/L
Serum Na	141	135–150 mEq/L
Serum K	3.6	3.5–5.3 mEq/L
Serum Cl	104	98–110 mEq/L
Serum Ca	9.8	8.8–10.2 mg/dL
Serum P	3.2	2.7–4.6 mg/dL
Serum Mg	2.1	1.8–2.3 mg/dL
CRP	0.37	<0.30 mg/dL
SARS-CoV-2 antigen	Negative	Negative
Urine test		
Leukocyte	Negative	Negative
Nitrite	Negative	Negative
Protein	Negative	Negative
Glucose	Negative	Negative
Urobilinogen	Normal	
Bilirubin	Negative	Negative
Ketone	Negative	Negative
Blood	Negative	Negative
pH	7.0	

**Figure 1:**
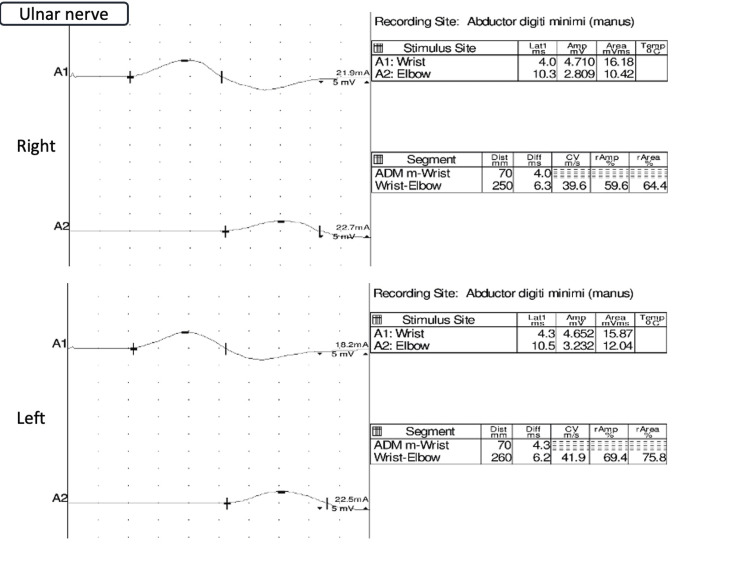
Nerve conduction velocity tests of ulnar nerves revealing possible demyelination and axonal injury.

**Figure 2:**
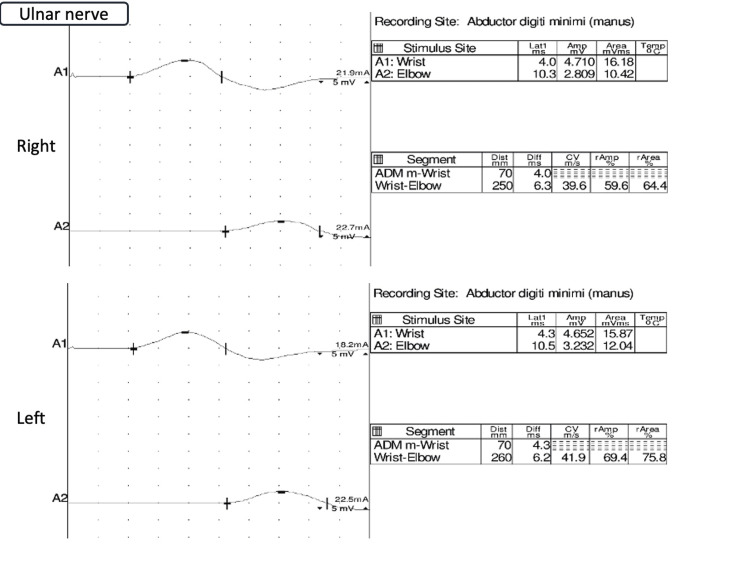
Nerve conduction velocity tests of ulnar nerves revealing possible demyelination and axonal injury.

Cerebrospinal fluid analysis revealed protein at 45 mg/dL, glucose at 97 mg/dL, cell count at 5/μL, and chloride at 123.7 mEq/L, with no albuminocytological dissociation observed. Magnetic resonance imaging of the head revealed no evident infarcts, hemorrhages, or inflammatory changes. Nerve conduction velocity testing of the radial nerves revealed possible demyelination and axonal injury (Figure 1), similar to that of the ulnar nerves (Figure 2).

Considering clinical progression, acute inflammatory demyelinating polyneuropathy was suspected. Intravenous immunoglobulin (Ig) therapy was initiated at a dose of 22.5 g/day (based on a body weight of 57 kg) and was continued for five days.

On the second day of hospitalization, lower limb paralysis, upper limb muscle weakness, and hoarseness were observed. On day 3, the patient’s condition progressed to quadriplegia. Owing to the respiratory muscle paralysis that led to respiratory distress, the patient was intubated and mechanically ventilated. No further progression of neurological symptoms was observed on day 3, and facial and neck muscle functions and eye movements were intact. Serological tests performed on admission revealed elevated Mycoplasma pneumoniae antibodies at a titer of 1:40 on day 5 of hospitalization, suggesting a preceding infection. Antinuclear antibodies, antineutrophil cytoplasmic antibodies, and anti-GQ1b IgG and anti-GM1 IgG antibodies yielded negative results.

**Figure 3:**
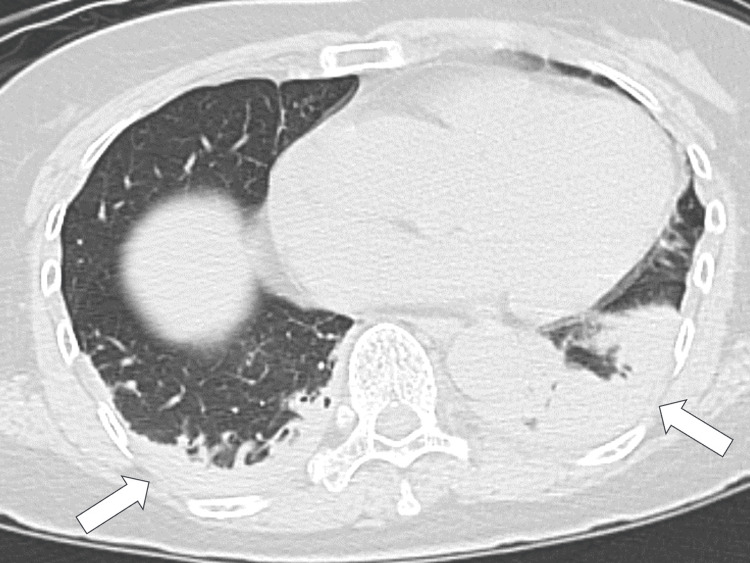
Chest computed tomography revealing bilateral infiltrations on the chest, indicating bilateral persistent pneumonia (white arrows).

On day 3 of hospitalization, her sputum became purulent, and Gram staining revealed a mixture of Gram-positive and Gram-negative bacteria. Chest radiography revealed infiltration of the right lower lobe, and the patient was diagnosed with aspiration pneumonia. Ampicillin-sulbactam was administered at a dose of 12 g/day. On day 5, she developed a fever of 37 °C. Owing to the possibility of ventilator-associated pneumonia, ampicillin-sulbactam was discontinued on day 6 and replaced with piperacillin-tazobactam at a dose of 13.5 g/day. Despite these interventions, she continued to have a fever and demonstrated decreased consciousness. On day 8, when a catheter-related bloodstream infection was suspected, the central venous catheter inserted on the third day was removed. The tips of the removed catheters and blood cultures were sent for microbiological analysis, and vancomycin was administered at a dose of 1 g/day. Cultures from the catheter tip and blood samples collected on day 8 yielded methicillin-resistant *S. epidermidis*, which was also identified in the sputum smear. Additionally, *Citrobacter koseri* was detected in sputum cultures on day 6. Chest computed tomography revealed bilateral infiltration, indicating persistent bilateral pneumonia (Figure 3).

On day 11, piperacillin-tazobactam was discontinued, and minocycline was started at 200 mg/d. Considering the possibility of an intestinal bacterial infection due to intestinal edema, meropenem was initiated at a dose of 3 g/d.

Subsequently, the patient’s fever subsided, and her respiratory condition stabilized. Blood cultures obtained on days 7 and 14 of hospitalization were negative. Her condition improved, and a gastrostomy was performed on day 24. General physicians, nurses, therapists, and social workers collaborated intensively and comprehensively for effective patient care. On day 46, the patient was transferred to the rehabilitation ward for continued rehabilitation.

## Discussion

This case demonstrates that severe GBS can cause immobility and dysfunction of multiple organ systems, which can inactivate patient immunity and cause occult bacteremia in previously healthy individuals. While treating patients with GBS, clinicians should focus on the impairment of neurological functions and subsequent immunosuppression caused by the immobility and dysfunction of multiple organs.

GBS can lead to immunosuppression owing to the immobility and dysfunction of multiple organs. In the present case, the causative pathogen of the bacteremia was methicillin-resistant *S. epidermidis*. The patient developed immunodeficiency during the course of GBS. Although the patient had no history of immunodeficiency, transient immunosuppression was reported owing to decreased peripheral blood lymphocytes and helper/inducer T-cells during the acute phase of Mycoplasma pneumonia [6]. As the onset of GBS may be caused by immunodeficiency or changes in immune function, it may manifest during the period of immunosuppression caused by Mycoplasma infection [7].

Furthermore, during the acute phase of GBS, a decrease in CD4+ and CD25+ cells is observed [8]. This suggests an excessive autoimmune response and a decline in the defense functions in the body. Overlapping conditions may induce immunosuppression. Additionally, reports indicate a tendency for severe conditions such as sepsis in co-infection with Mycoplasma and bacteria, underscoring the importance of preventing secondary infections [9]. In our case, the M. pneumoniae antibody titer was 40-fold higher on the first day of illness, suggesting that M. pneumoniae infection persisted at admission. If co-infection with Mycoplasma and bacteria was a factor in the exacerbation, administering antibiotics against Mycoplasma infection might have reduced the risk of sepsis.

The immunosuppression of GBS can lead to various infections. One of the major outcomes of GBS is respiratory muscle paralysis, which necessitates mechanical ventilation [1]. Similar to the present case, the need for intubation and mechanical ventilation, combined with vocal cord paralysis, predisposed the patient to aspiration pneumonia. Mechanical ventilation has associated risks, notably ventilator-associated pneumonia, which can be life-threatening. This scenario does not represent direct immunosuppression but rather an increased vulnerability to infections owing to GBS complications. As observed in severe GBS cases, prolonged hospital stays increase the risk of hospital-acquired infections [3]. In this case, the patient had a central venous catheter, which later became a source of bacteremia because of a methicillin-resistant *S. epidermidis* infection. *C. koseri*, an intestinal bacterium, was also detected in sputum cultures. The presence of this bacterium in the respiratory system indicated possible aspiration owing to the paralyzed respiratory muscles and vocal cords. Extended bedridden periods can cause complications such as bedsores or deep vein thrombosis [10,11]. If not managed promptly, infections can result in complications.

General physicians' systems thinking is essential for managing GBS, including immunodeficiencies. The holistic management of GBS requires addressing the primary neurological deficits and secondary complications [12]. This mandates a systems-thinking approach in which each system of the body and its potential vulnerabilities are assessed [13]. Considering the myriad of complications that arise from GBS, a proactive approach can aid in preventing or minimizing these complications. For instance, understanding the risk of aspiration and employing preventive measures or being vigilant regarding potential sources of infection, such as catheters, can be lifesaving. The management of GBS requires coordinated efforts from various medical specialties. To ensure improved care for complicated patients, such as those in this case, various professionals need to collaborate closely to provide comprehensive care in rural community hospitals [14].

## Conclusions

GBS does not inherently cause immunosuppression; its severe manifestations can indirectly place patients in a state mimicking immunodeficiency. Recognizing this potential complication is crucial for a timely intervention. Adopting a systems-thinking approach allows physicians to anticipate, prevent, and manage these complications, thereby optimizing patient outcomes. The case of a 70-year-old woman underscores the need for a comprehensive multidisciplinary approach to managing GBS and its associated challenges.
